# Research Design, Protocol, and Participant Characteristics of COLEAFS: A Cluster Randomized Controlled Trial of a Childcare Garden Intervention

**DOI:** 10.3390/ijerph182413066

**Published:** 2021-12-10

**Authors:** Nilda Graciela Cosco, Nancy M. Wells, Muntazar Monsur, Lora Suzanne Goodell, Daowen Zhang, Tong Xu, Derek Hales, Robin Clive Moore

**Affiliations:** 1 Department of Landscape Architecture and Environmental Planning, College of Design, North Carolina State University, Raleigh, NC 27695, USA; robin_moore@ncsu.edu; 2 Department of Human Centered Design, College of Human Ecology, Cornell University, Ithaca, NY 14853, USA; tx66@cornell.edu; 3 Department of Landscape Architecture, College of Agricultural Sciences and Natural Resources, Texas Tech University, Lubbock, TX 79409, USA; mmonsur@ttu.edu; 4 Department of Food, Bioprocessing and Nutrition Sciences, College of Agriculture and Life Sciences, North Carolina State University, Raleigh, NC 27695, USA; lsgoodel@ncsu.edu; 5 Department of Statistics, College of Sciences, North Carolina State University, Raleigh, NC 27695, USA; dzhang2@ncsu.edu; 6 Department of Nutrition, Gillings School of Global Public Health, University of North Carolina-Chapel Hill, Chapel Hill, NC 27599-7400, USA; derekh@email.unc.edu

**Keywords:** children, childcare, gardening, randomized controlled trial, healthy eating, physical activity

## Abstract

Childcare garden interventions may be an effective strategy to increase fruit and vegetable (FV) consumption and physical activity among young children. The objective of this paper is to describe the research design, protocol, outcome measures, and baseline characteristics of participants in the Childcare Outdoor Learning Environments as Active Food Systems (“COLEAFS”) study, a cluster randomized controlled trial (RCT) examining the effect of a garden intervention on outcomes related to diet and physical activity. Fifteen childcare centers in low-income areas were randomly assigned to intervention (to receive garden intervention in Year 1), waitlist control (to receive garden intervention in Year 2), and control group (no intervention). The garden intervention comprised six raised beds planted with warm-season vegetables and fruits, and a garden activity booklet presenting 12 gardening activities. FV knowledge and FV liking were measured using a tablet-enabled protocol. FV consumption was measured by weighing FV before and after a snack session. Physical activity was measured using Actigraph GT3x+ worn by children for three consecutive days while at the childcare center. Of the 543 eligible children from the 15 childcare centers, 250 children aged 3–5 years received parental consent, assented, and participated in baseline data collection. By employing an RCT to examine the effect of a garden intervention on diet and physical activity among young children attending childcare centers within low-income communities, this study offers compelling research design and methods, addresses a critical gap in the empirical literature, and is a step toward evidence-based regulations to promote early childhood healthy habits.

## 1. Introduction

Young children often fall short of recommended levels of daily fruit and vegetable (FV) consumption and physical activity (PA) in the United States [1]. Children aged 2–4 years score 61 out of 100 on the USDA’s healthy eating index (“HEI”) [2]. Among children aged 2–3 years, 52% do not meet recommended daily consumption of fruit and vegetables (FV) (2–3 cups), while among those aged 4–6 years, more than 90% of children do not meet FV consumption recommendations [3]. Children aged 3–5 years should be physically active for 3 h per day, which is equivalent to 15 min per hour [4]. However, approximately 42% of preschool age boys and 60% of preschool age girls do not meet these recommendations [4]. Inadequate FV consumption and physical activity are particular concerns, as the prevalence of obesity among children remains a significant public health issue in the United States [5], especially for those living in lower income households [6,7].

School gardens have been recognized as a strategy to promote healthy dietary intake and physical activity among youth. Research suggests that gardening may encourage children’s interest in and acceptance of FV by directly engaging them in micro-scale food production [8,9,10]. While little research has examined the effects of school gardens on children’s PA, extant evidence suggests that gardens may increase PA and reduce sedentary time among children aged 8–12 years [11]. However, studies of school gardens have typically focused on elementary school age children, approximately 6–10 years old [12,13], with little study of pre-school children 3–5 years of age. Moreover, few cluster randomized controlled trials have been conducted [11,14,15,16].

This study is grounded in two conceptual models. First, the life course perspective informs the focus on early childhood, a time when the foundation is laid for life-long habits [17,18,19]. Introducing young children to FV gardening may help to set them on positive life course trajectories toward physical activity [11] and healthy eating [8]. Second, this research was shaped by the ecological model, which indicates that development and behavior are influenced by the direct and interactive effects of “microsystems”—or settings—in which we live, learn, work, and play, in tandem with multi-level factors associated with culture, norms, and policies [20,21]. Childcare environments are among the most influential settings for very young children [22].

In 2019, 61% of children aged 3–5 years in the U.S. attended childcare [23]. As reliance on childcare has increased in recent decades [22], evidence has emerged suggesting that interventions within childcare settings can affect children’s health-related behaviors [24]. However, there is a paucity of studies targeting childcare centers [25,26], and empirical evidence related to the effects of gardening on the diet and physical activity of young children is particularly scant, indicating a need for more research to examine intervention effectiveness.

The Childcare Outdoor Learning Environments as Active Food Systems (“COLEAFS”) study is based on a field-tested outdoor renovation strategy [27] operational since 2006, conducted with an established network of childcare center partners including an FV garden installation protocol for 3–5-year-old children. The long term objectives of the overall COLEAFS project are to: (1) improve the health of vulnerable preschool children attending U.S. childcare centers by increasing physical activity (PA) and consumption of fresh fruit and vegetables (FV); (2) influence childcare regulations to include hands-on FV gardening as a health promotion policy, potentially impacting regulated U.S. childcare centers; and (3) increase awareness and understanding of early childhood physical activity and fruit and vegetable eating through hands-on FV gardening for educators, parents, Cooperative Extension agents, and Quality Rating and Improvement Systems (QRIS) operating in all states, District of Columbia, and some U.S. territories [28].

The goal of this randomized controlled trial is to examine the impact of the FV garden component of COLEAFS on fruit and vegetable (FV) liking, identification, consumption, and physical activity, within an understudied population—children 3–5 years old in under-resourced communities [29]. This paper describes the research design, protocol, outcome measures, and baseline characteristics of study participants.

## 2. Materials and Methods

### 2.1. Study Design

**Figure 1:**
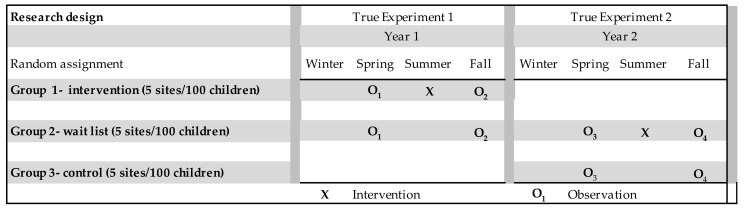
Research design with intervention, waitlist (delayed intervention), and control groups. Note: “fall” data collection was conducted by late summer to avoid attrition of “graduating” five-year-old children.

Fifteen childcare centers were randomly assigned to one of three groups: intervention (“Group 1,” *n* = 5 centers, ~100 children); waitlist control (or “delayed intervention”) (“Group 2,” *n* = 5 centers, ~100 children); and no intervention control (“Group 3,” *n* = 5 centers, ~100 children). As illustrated in Figure 1, in Year 1 (Spring), baseline data were collected from Groups 1 and 2. Group 1, the initial intervention centers, then received the garden intervention in the summer of Year 1 and both groups were assessed in the fall, post intervention. No further data were collected from Group 1. In Year 2, Groups 2 and 3 were assessed in the spring. Group 2, the waitlist control centers, received the garden intervention in the summer of Year 2, and both Groups 2 and 3 were assessed in the fall of Year 2. The study is registered with ClinicalTrials.gov #NCT04864574. The research design and methods were approved by the North Carolina State University Institutional Review Board (IRB), protocol approval #5908.

### 2.2. Childcare Centers, Participants, and Recruitment

#### 2.2.1. Center Recruitment and Randomization

Childcare centers were identified in collaboration with Wake County Smart Start, North Carolina, U.S.A. from a pool of 310 licensed centers in the county with ratings of four or five (out of five) stars [30]. Table 1 summarizes the center-level study eligibility criteria.

Wake County Smart Start disseminated invitations to eligible centers to participate in the project. Invitations included a project description, incentive details, and the application URL.

To apply, centers completed an online application that included verification of selection criteria (Table 1) and a statement of willingness to work collaboratively with the research team, including attending a short briefing session conducted at the center. At each prospective center, a minimum of two preschool teachers that were committed to conducting FV gardening on-site were identified. These teachers agreed to participate in two, 2-week data-gathering periods, and, if randomly assigned to the intervention, to complete a gardening record (Garden Activity Fidelity Tool, “GAFT”) each week during intervention period, engage in regular journaling of classroom activities, and record anecdotal data. For centers submitting a complete application, physical site boundaries were determined, and the site was assessed using Wake County GIS resources, Google Earth, and on-site inspection. Sites with insufficient sun exposure (i.e., less than 6 h/day of full sun) and/or inadequate service access (e.g., lack of water source, lack of space, shared space with incompatible use) were excluded.

From the centers that met the selection criteria, 15 were randomly selected to participate in the study. The remaining applicant centers served as a back-up pool. Meetings were conducted with center directors and designated preschool teachers to review project aims and expectations, and to re-confirm willingness to collaborate. The 15 selected centers were then randomly assigned to Groups 1, 2, and 3. Because the selected childcare centers vary substantially regarding the percentage of families that received subsidies, random assignment was “balanced” to ensure the distribution of percent subsidization within each of the three groups. (The 15 centers were divided into five groups of three that had approximately the same percentage of subsidization. Random numbers were generated for each center. Then, within each group, each center received a ranking (1, 2, 3) based on its random number. Centers were assigned to Groups 1, 2, and 3 based on within group ranking.) Once identified, the intervention centers signed a letter of agreement specifying incentives: full garden installation (approximate value $1500), weekly garden support during growing season, teacher incentives (up to $250/individual if involved in all data-gathering periods), and gardening resources.

#### 2.2.2. Child Recruitment

Participants were 3–5-year-old children enrolled in the selected 15 childcare centers. Parents received an invitation to enroll their children in the study one month prior to the scheduled data-gathering session. The invitation contained a description of the study in lay language and two illustrative images: (1) children gardening with a teacher, (2) the research assistant securing an accelerometer on a child’s waist.

Teachers collected consent forms from parents and a research assistant gathered them weekly. Children without parental consent were identified by the teacher and invited to join an activity in a different space within the center during data collection (e.g., adjacent classroom or playground). No data were collected from children whose parents did not consent for them to participate. Children also assented to participate. On the data collection days, children were invited to participate and asked if they were ready to start. Children who did not want to participate in the research tasks planned for the day continued their habitual activities in the classroom.

### 2.3. Intervention Components

**Figure 2:**
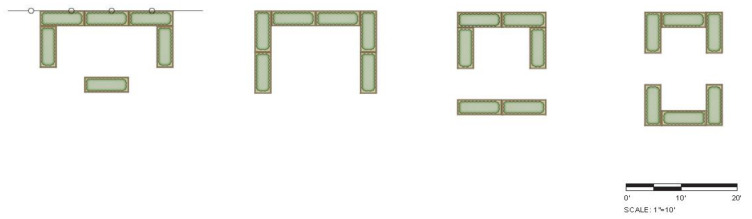
Four possible raised bed garden configurations allow for working from most sides and in-ground planting of annual vegetables and perennial fruiting plants.

The intervention comprised garden installations, resources, and gardening activities. Gardens included six raised planting beds, 8 feet × 2 feet and 10” high, installed outdoors. Raised beds were located within convenient access from the classroom and configured in one of four standard layouts according to physical site conditions (Figure 2) to ensure that they received direct sun at least eight hours per day, were close to a hose bib, had sufficient space to allow for working from most sides and in-ground planting of annual vegetables and perennial fruiting plants.

A coffer storage unit (Suncast 50-gallon deck box) and a garden kit (hand tools, hose, gardening gloves, watering wand, and child-sized cans) were provided. Each raised planting bed was constructed from a standard kit of parts, installed by the research team. Beds were placed parallel to a south-facing wall of childcare center buildings or parallel to fence lines meeting maximum sun exposure. Space at each end of the planters was designated as vegetable vine spillover space, surfaced with straw. Planting beds were filled with high quality, well-drained, moisture-retentive growing medium.

Fruits and vegetable selection criteria were: (a) documented record of warm season harvest success in the North Carolina Piedmont region, (b) able to be eaten raw, (c) harvest times occurring during the same overall warm growing season, (d) an equal number of nutritious fruits and vegetables. Fruit trees were not selected because of multi-year time required to produce.

**Figure 3:**
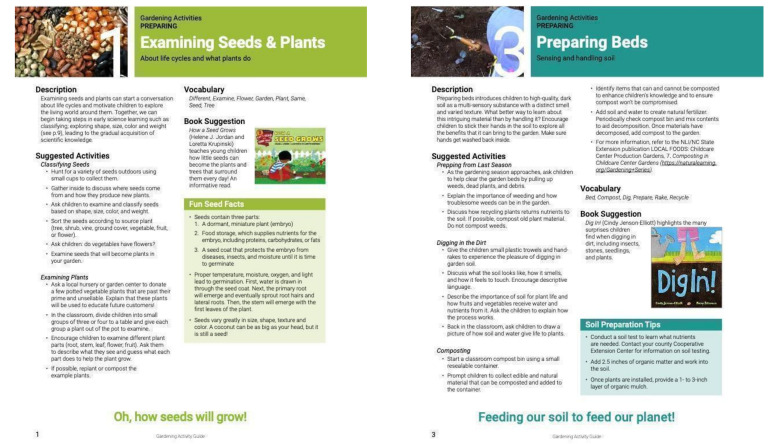
Gardening Activity Guide sample pages.

“The Garden Activity Guide” was a colorful, detailed booklet that provided 12 age-appropriate, seasonally prescribed activities (Figure 3). The 12 gardening activities, led by the teacher, fell under three themes: preparing, caring, and harvesting/eating. PREPARING included: (1) examining seeds and plants, (2) sprouting seeds, (3) preparing beds, (4) planting; CARING included: (5) watering, (6) weeding, (7) observing plant growth, (8) observing garden bugs; HARVESTING/EATING included: (9) harvesting, (10) preparing, (11) snacking, (12) taking home. Teachers received a one-on-one orientation to the activity guide to ensure that they were prepared to lead all 12 activities. In addition, each week, the RA visited the classroom to interview teachers, discuss challenges and successes of the prior week, and provide technical assistance.

### 2.4. Constructs and Measures

Demographic variables including age, gender, parental education, race/ethnicity, special needs, and height/weight (BMI) were collected at baseline by RAs. Weight was measured in grams using a digital scale (Tanita HD-357). Children’s height was measured to the closest centimeter using a portable stadiometer (Hopkins Medical Products Portable Stadiometer, Model Number 680214). Body mass index (BMI) was calculated using the CDC BMI-for-age charts [31].

Dependent variables included (1) fruit and vegetable (FV) identification, (2) FV liking, (3) FV consumption, and (4) min of sedentary, light, and moderate/vigorous physical activity. In addition, teachers recorded activities and anecdotal data on the Garden Activity Fidelity Tool (GAFT) chart each week during intervention periods. All measures used in this study had established reliability and validity for use with children aged 3–5 years.

#### 2.4.1. FV Identification and FV Liking

**Figure 4:**
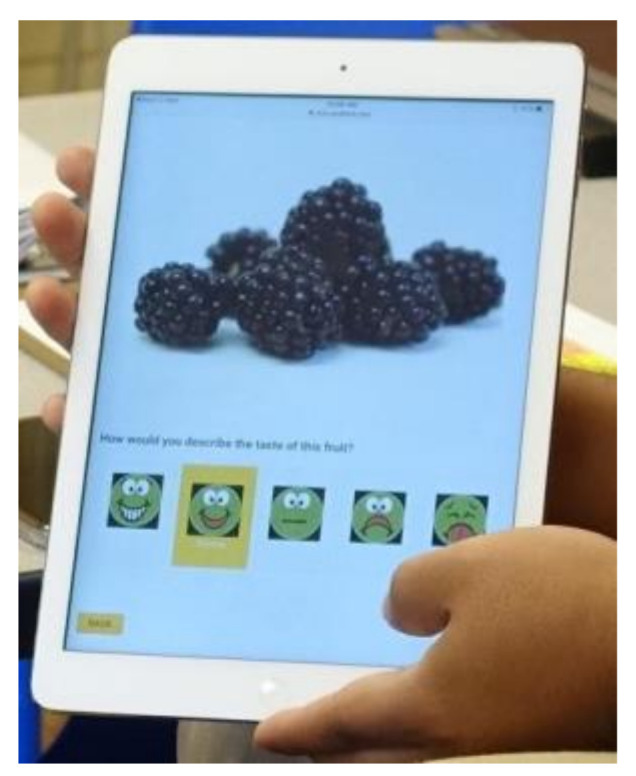
Modified “super yummy/super yucky” electronic recording system.

FV identification and FV liking were measured using a modified electronic method (“super yummy/super yucky”) developed to assess fruit and vegetable (FV) liking among children aged 3–5 years [32]. The modified electronic version (Figure 4) used a digital picture-based survey system on touch-enabled tablets, replicating the original paper-based instrument, containing 20 high-quality, digital photographs appropriate for digital devices. Included were six fruits, six vegetables, and practice images showing a consistent look without adjacent objects (e.g., plate, cup) [32]. The electronic modification was intended to eliminate data entry steps, reduce entry errors, and facilitate file conversion for statistical analysis. This method yielded three identification variables and three liking variables—i.e., fruit only (F), vegetables only (V), and both FV.

The original measure demonstrated strong internal consistency (alpha = 0.79) [32]. Test-retest reliability (with 7–14 days between tests) was acceptable for both the 9-item fruit scale (*r_s_* = 0.51), the 10-item vegetable scale (*r_s_* = 0.40), and the combined FV scale (*r_s_* = 0.49). Predictive validity was established based on Wilcoxon sign-ranked test for differences between the FV liking ratings based on photos and “taste and rate” assessments [32].

**Figure 5:**
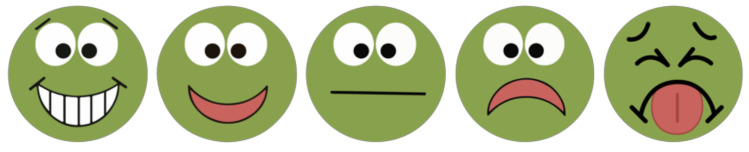
Non-gendered 5-point face scale “super yummy/super yucky”.

A day before the FV identification and FV liking measure was presented to children, they participated in a storytelling training session conducted by research assistants. The story “Plucky the Pea” illustrated the pictorial scale showing five rating options (Figure 5). Before data gathering, research assistants reminded the group of participating children of the story and reviewed the rating scale before working with them individually.

FV Identification was measured by asking the child if he/she knows (Y/N) each of the six fruits and six vegetables as shown, in turn, on the tablet screen. The research assistant (RA) then asked the child to name each item. RAs were instructed to record all answers phonetically including incorrect names as spoken by the child.

FV liking was measured by asking the child to point at a non-gendered 5-point face scale (super yummy/super yucky) to determine level of liking for each fruit and vegetable (Figure 5) where 5 = super yummy, 4 = yummy, 3 = just okay, 2 = yucky, 1 = super yucky.

#### 2.4.2. FV Consumption

FV consumption was measured using the Fruit and Vegetable Snack Tool (F&VST)—a modified version of the measure developed by Witt and Dunn (2012) to operationalize FV consumption among children aged 3–5 years [33]. Before snack time, store-bought, standard pieces of vegetables and fruit prepared in the lab were offered on two consecutive days, as follows: Day 1, six vegetables: cucumber, green bean, red pepper, yellow squash, tomato, and zucchini. Day 2, six fruits: apple, blueberries, blackberry, strawberry, cantaloupe, and watermelon. Vegetables and fruits were offered on individual trays (6” x 12”) containing 6 cups (approximately 50 g/each) labeled with a child’s name and ID number. All fruit and vegetables were weighed on a scale in grams before and after consumption sessions, including food touched or wasted by the child. Uneaten food was composted. This protocol yielded both grams consumed, and percentage consumed for F, V, and FV combined. Fruits and vegetables offered for consumption were the same as shown in the FV identification and FV liking tablet measure and planted in the intervention gardens except for apples.

#### 2.4.3. Physical Activity

Physical activity was measured using accelerometry, which is a valid, objective approach to measuring physical activity among preschool-age children [34]. Children wore Actigraph GT3x+ accelerometers for three consecutive days during childcare hours at each wave of data collection. Accelerometers were attached to static nylon belts. Trained research staff attached accelerometers to each child on the morning of the first data collection day. Belts were worn around the waist with monitors over the right hip during all childcare activities. At the end of each day, monitors were removed. The monitors were labeled so that the child wore the same monitor each day. Research staff logged on and off times for each participant and recorded the start and end of nap time each day. After data collection was complete, data were downloaded and converted to 5 s epoch-level files using ActiLife software (ActiGraph, Pensacola, FL, USA). Non-wear, wear, and sleep (nap) were assigned using the Choi algorithm, data collection logs, custom algorithms, and visual inspection of the data [35]. Days with 270 min of waking wear were considered complete (~75% of childcare day). Most days are expected to have at least 360+ min of wear. For each “good” day, standard cutpoints were used to determine minutes of sedentary (<8.3 counts/5 s), light (8.4–191 counts/5 s), moderate (192–334 counts/5 s), and vigorous (≥335 counts/5 s) physical activity. Overall activity was estimated using total counts per day.

#### 2.4.4. Garden Fidelity Tool

**Figure 6:**
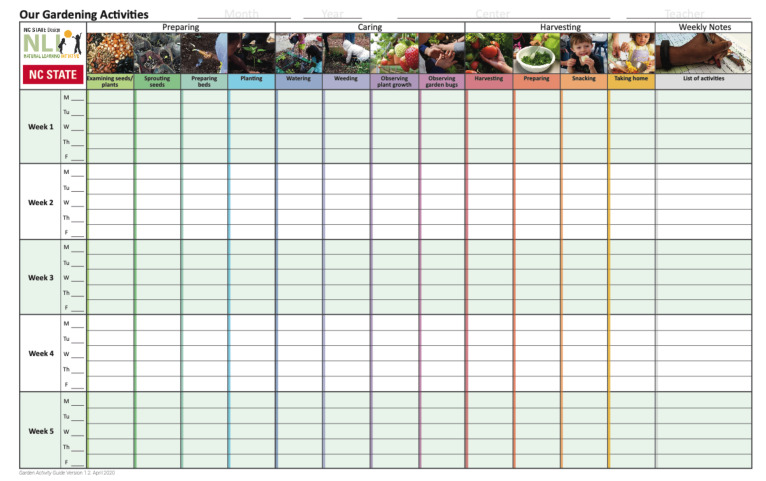
GaFT laminated wall chart to record gardening activities.

Garden fidelity was measured using the Garden Fidelity Tool (GaFT) to capture variations between garden interventions from center to center (Figure 6). GaFT measured exposure of the preschool children to the seasonally prescribed program of FV gardening experiences. GaFT was piloted in 2014 in 8 childcare centers receiving the environmental intervention, including the warm season gardening component. COLEAFS data were gathered daily by teachers during intervention periods. GaFT employs a 11” x 17” laminated wall chart with “stickers” in respective cells (identified by gardening activities across top, months/days left column), and weekly notes on the right column, mounted in a designated location visible to children (Figure 6). During morning “circle time”, the teacher discussed with children if a gardening activity occurred the previous day; if so, stickers were added to appropriate cell(s). The maximum score was five stickers/week/activity for a total weekly maximum of 60. Each month, RAs photographed charts, archived records, removed stickers, returned charts, and entered data into the GaFT spreadsheet.

### 2.5. Analytic Strategy

Descriptive statistics for demographic variables will be presented by frequency or percentage (categorical variables) and as means and standard deviations (for continuous variables). Descriptive statistics for control variables (i.e., environment quality) and key outcome variables (F, V, FV liking, F, V, FV identification, F, V, FV consumption, and physical activity) will be summarized by mean and standard deviation or median and inter-quartile range.

Formal statistical analyses addressing the four research questions (i.e., the effects of childcare center FV garden intervention on children’s (1) FV identification, (2) FV liking, (3) FV consumption, and (4) physical activity), will employ linear mixed modeling via MIXED procedure of SAS software (v 9.4) (SAS Institute, Inc, Cary, NC, USA), which takes into account the nested structure of the data given the fact that the unit of analysis is the childcare center and each child’s longitudinal data are nested within centers.

We conducted a power analysis for each primary outcome at level 0.05 at various effect sizes. The power analysis, based on the variance estimates for centers, subjects, and residual errors from the current data, indicated that a sample of 15 centers with 250 children would yield powers of 0.87–0.91 and effect sizes of 1 to 6 for the four outcome variables.

## 3. Results

### Recruitment and Enrollment

**Figure 7:**
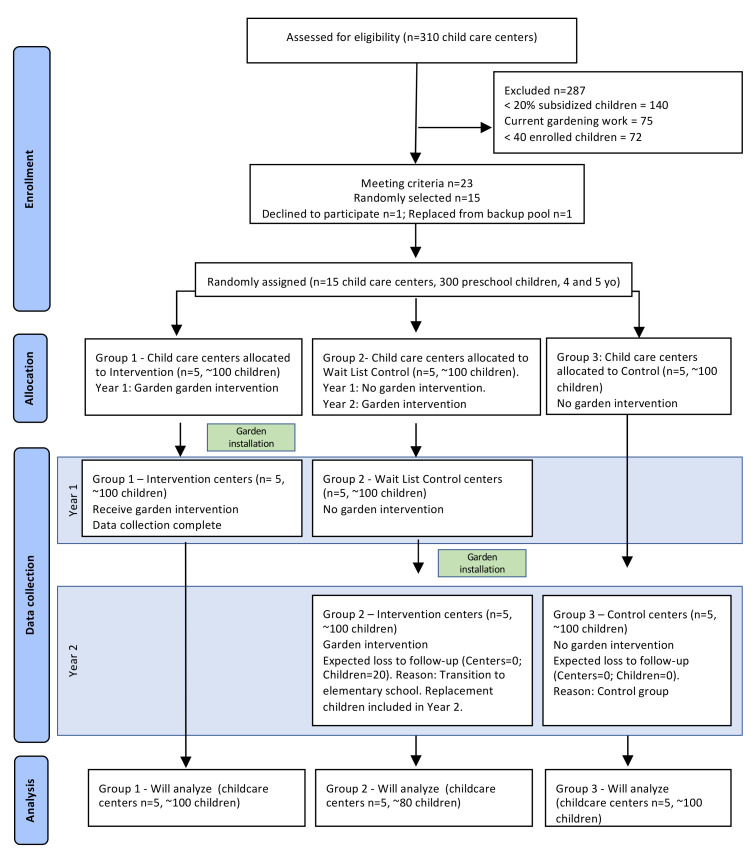
COLEAFS CONSORT diagram.

Recruitment and enrollment are illustrated in the CONSORT diagram (Figure 7). The list of 310 licensed centers in Wake County, NC was secured, and centers were assessed for eligibility. Twenty-three centers were identified as meeting the inclusion criteria (Table 1). From the 23 centers that met the selection criteria, 15 were randomly selected to participate in the study. The remaining eligible childcare centers served as a back-up pool. One center withdrew and was replaced from the back-up pool.

Of the 543 eligible 3–5-year-old children attending the 15 selected childcare centers, 250 children were consented by parents to participate.

#### Characteristics of the Randomized Groups

Characteristics of the randomized groups were as follows. *Intervention:* 61 children, mean child age, 3.17 years; 50.80% male; 58.90% non-white; 44.30% receiving subsidies; and mean BMI 16.13. *Waitlist* (delayed intervention): 119 children, mean child age, 3.15 years; 44.90% male; 62.20% non-white; 47.90% receiving subsidies; and mean BMI 16.20. *Control*: 70 children, mean child age, 3.51 years; 53.60% male; 71.90% non-white; 62.30% receiving subsidies; and mean BMI 15.97. Non-white children included African American, Asian, Latino, and Multi-racial (Table 2). Participating children were at healthy weight showing similar BMI mean results by group (Table 2).

Center recruitment can be a challenge within low-income communities. Although the original intention was that within all the participating childcare centers, 50% or more families served would receive childcare subsidies, this was not possible. In some of the childcare centers serving a high percentage of subsidized families, the childcare provider served also as the cook, the accountant, the driver, etc., and is simply too taxed to make time to partner in a research study. Therefore, centers with lower percentages of enrollment eligible for subsidies and willing to participate in the study were identified in collaboration with the Wake County Smart Start. Given this reality, subsidy percentages in our sample ranged from 12% to 95%, with 8 of the 15 centers having a clientele comprising 50% or more of families receiving subsidies. Fortunately, this variability was taken into account via the random assignment. The waitlist group was larger than other groups because children participated in two consecutive years (as control in year 1 and as intervention in year 2), and incoming children were recruited to make up for those transitioning to elementary school.

## 4. Discussion

The COLEAFS RCT targeted 15 childcare centers within Wake County, North Carolina. By examining children within low-income communities, the study addressed a population that is particularly vulnerable to poor dietary intake, physical inactivity, and ultimately, obesity [5]. Moreover, by studying young children (ages 3–5 years) within low resource communities, this research examined an under-studied group [29] and will provide insight to the efficacy of early-life interventions on health behaviors.

This study is the first 2-year RCT to examine the effects of a garden intervention within childcare centers. The longitudinal design—over 2 years—addresses criticism of many past studies examining the effects of garden interventions on diet and physical activity that were of relatively brief duration. In addition to being a true experiment (RCT) with a longitudinal research design, the study has several unique strengths. Use of a waitlist control (delayed intervention) group (Group 2) that serves as control in year 1 and as intervention in year 2 allows for a replication in years 1 and 2. Moreover, the internal validity of this study—i.e., our capacity to make causal inferences—is strengthened by the research design that enabled the examination of multiple (three) “pre-test” (pre-intervention) measures of outcomes in Group 2 (as illustrated in Figure 1). Multiple pre-tests allow for the examination of change over time *prior* to the intervention in order to rule out several potential alternative explanations—i.e., threats to internal validity, such as history, testing, maturation, and regression to the mean that are seldom addressed in studies of this type [36]. Valid, reliable, age-appropriate measures were used for all four dependent variables. When possible, constructs were measured objectively (e.g., accelerometers used to measure physical activity). Moreover, this study included a measure of garden activity fidelity (GAFT) (elsewhere referred to as “garden intervention fidelity” (GIF) [16,37]. By operationalizing the robustness of the garden intervention, the GAFT allows for the examination of dose-response relation and builds upon prior studies that have found garden fidelity to be an important predictor of an impact on fruit and vegetable consumption [12,37,38].

Because childcare centers are policy-sensitive institutions, evidence determining the benefits of FV gardening may encourage regulators to adopt supportive rules [39]. With most of the U.S. population living in areas with long annual growing seasons [40], gardening may be a promising obesity prevention strategy for young children in those regions where most U.S. regulated childcare centers are located [41].

## 5. Conclusions

The COLEAFS randomized controlled trial examined the effects of a childcare garden intervention on FV identification, FV liking, FV consumption, and physical activity among preschool children aged 3–5 years. Findings from this study may inform the implementation of fruit and vegetable gardens within childcare centers to promote healthy dietary intake, foster physical activity, and contribute to obesity prevention among young children.

## Figures and Tables

**Table 1:** Childcare Center Eligibility Criteria.

Childcare Center Elegibility Criteria
(1) Assigned a 4 or 5 Star Rated License, NC Division of Child Development and Early Education
(2) Serve a majority of children eligible for the childcare subsidy programs
(3) Contain at least two preschool classrooms (3–5-year-old children)
(4) Commitment from two preschool teachers to support recruitment and data gathering
(5) Enrollment size within the middle third for Wake Co (excluding smallest + largest centers)
(6) Operate a regulated on-site kitchen to prepare food for snacks
(7) Employ cooking staff
(8) Operate a year-round calendar
(9) Own or lease current space for at least 5 years into the future
(10) Do not currently conduct on-site FV gardening but interested in implementing in the future

**Table 2:** COLEAFS center-level characteristics at baseline, by intervention (I), waitlist (delayed intervention) (W), and control centers (C).

Group	n	Age $\bar{x}$ (sd)	BMI $\bar{x}$ (sd)	% Male	% Non-White	% Subsidy
Intervention	61	3.17 (0.53)	16.13 (1.31)	50.80%	58.90%	44.30%
Waitlist	119	3.15 (0.55)	16.20 (1.63)	44.90%	62.20%	47.90%
Control	70	3.51 (0.56)	15.97 (1.27)	53.60%	71.90%	62.30%

## Data Availability

The data presented in this study are available on request from the corresponding author. The data are not publicly available due to the study focus on young children.

## References

[1] Tagtow A., Rahavi E., Bard S., Stoody E.E., Casavale K., Mosher A. (2016). Coming together to communicate the 2015–2020 dietary guidelines for Americans. J. Acad. Nutr. Diet..

[2] U. S. Department of Agriculture, U. S. Department of Health and Human Services Dietary Guidelines for Americans, 2020–2025.

[3] Guenther P.M., Dodd K.W., Reedy J., Krebs-Smith S.M. (2006). Most Americans eat much less than recommended amounts of fruits and vegetables. J. Am. Diet. Assoc..

[4] Pate R.R., O’Neill J.R., Brown W.H., Pfeiffer K.A., Dowda M., Addy C.L. (2015). Prevalence of compliance with a new physical activity guideline for preschool-age children. Child. Obes..

[5] Cunningham S.A., Kramer M.R., Narayan K. (2014). Incidence of childhood obesity in the United States. N. Engl. J. Med..

[6] Lowry Warnock A., Dooyema C., Blanck H.M., Lee S.H., Hall K., Geary N., Galuska D.A. (2021). A Healthy Start: National Trends in Child Care Regulations and Uptake of Obesity Prevention Standards (2010–2018). Child. Obes..

[7] Ogden C.L., Carroll M.D., Fakhouri T.H., Hales C.M., Fryar C.D., Li X., Freedman D.S. (2018). Prevalence of obesity among youths by household income and education level of head of household—United States 2011–2014. Morb. Mortal. Wkly. Rep..

[8] Cabalda A.B., Rayco-Solon P., Solon J.A.A., Solon F.S. (2011). Home gardening is associated with Filipino preschool children’s dietary diversity. J. Am. Diet. Assoc..

[9] Davis J.N., Spaniol M.R., Somerset S. (2015). Sustenance and sustainability: Maximizing the impact of school gardens on health outcomes. Public Health Nutr..

[10] Meinen A., Friese B., Wright W., Carrel A. (2012). Youth Gardens Increase Healthy Behaviors in Young Children. J. Hunger Environ. Nutr..

[11] Wells N.M., Myers B.M., Henderson C.R. (2014). School gardens and physical activity: A randomized controlled trial of low-income elementary schools. Prev. Med..

[12] Christian M.S., Evans C., Nykjaer C., Hancock N., Cade J. (2014). Evaluation of the impact of a school gardening intervention on children’s fruit and vegetable intake. A randomised controlled trial. Int. J. Behav. Nutr. Phys. Act..

[13] Landry M.J., van den Berg A.E., Hoelscher D.M., Asigbee F.M., Vandyousefi S., Ghaddar R., Jeans M.R., Waugh L., Nikah K., Sharma S.V. (2021). Impact of a School-Based Gardening, Cooking, Nutrition Intervention on Diet Intake and Quality: The TX Sprouts Randomized Controlled Trial. Nutrients.

[14] Davis J.N., Nikah K., Asigbee F.M., Landry M.J., Vandyousefi S., Ghaddar R., Hoover A., Jeans M., Pont S.J., Richards D. (2019). Design and participant characteristics of TX sprouts: A school-based cluster randomized gardening, nutrition, and cooking intervention. Contemp. Clin. Trials.

[15] Evans C.E., Ransley J.K., Christian M.S., Greenwood D.C., Thomas J.D., Cade J.E. (2013). A cluster-randomised controlled trial of a school-based fruit and vegetable intervention: Project Tomato. Public Health Nutr..

[16] Wells N.M., Myers B.M., Todd L.E., Barale K., Gaolach B., Ferenz G., Aitken M., Henderson C.R., Tse C., Pattison K.O. (2015). The effects of school gardens on children’s science knowledge: A randomized controlled trial of low-income elementary schools. Int. J. Sci. Educ..

[17] Elder G.H., Damon W., Lerner R.M. (1998). The life course and human development. Handbook of Child Psychology.

[18] Wethington E. (2005). An overview of the life course perspective: Implications for health and nutrition. J. Nutr. Educ. Behav..

[19] Wheaton B., Gotlib I.H., Gotlib I.H., Wheaton B. (1997). Trajectories and turning points over the life course: Concepts and themes. Stress and Adversity Over the Life Course.

[20] Bronfenbrenner U., Morris P., Damon W., Lerner R.M. (1998). The ecology of developmental process. Handbook of Child Psychology.

[21] Sallis J.F., Cervero R.B., Ascher W., Henderson K.A., Kraft M.K., Kerr J. (2006). An Ecological Approach to Creating Active Living Communities. Annu. Rev. Public Health.

[22] Story M., Kaphingst K.M., French S. (2006). The role of child care settings in obesity prevention. Future Child..

[23] National Center for Education Statistics Enrollment Rates of Young Children. https://nces.ed.gov/programs/coe/indicator/cfa.

[24] Robinson-O’Brien R., Story M.T., Heim S. (2009). Impact of Garden-Based Youth Nutrition Intervention Programs: A Review. J. Am. Diet. Assoc..

[25] Larson N., Ward D.S., Neelon S.B., Story M. (2011). What role can child-care settings play in obesity prevention? A review of the evidence and call for research efforts. J. Am. Diet. Assoc..

[26] Waters E., de Silva-Sanigorski A., Burford B.J., Brown T., Campbell K.J., Gao Y., Armstrong R., Prosser L., Summerbell C.D. (2011). Interventions for preventing obesity in children. Cochrane Database Syst. Rev..

[27] Cosco N.G., Moore R.C., Smith W.R. (2014). Childcare outdoor renovation as a built environment health promotion strategy: Evaluating the preventing obesity by design intervention. Am. J. Health Promot..

[28] National Center on Early Childhood Quality Assurance QRIS Resource Guide; U.S. Department of Health and Human Services, Administration for Children and Families. https://ecquality.acf.hhs.gov/resource-guide.

[29] Hollar D., Messiah S., Lopez-Mitnik G., Hollar T., Almon M., Agatston A. (2011). Effect of a Two-Year Obesity Prevention Intervention on Percentile Changes in Body Mass Index and Academic Performance in Low-Income Elementary School Children. Am. Public Health Assoc..

[30] Division of Child Development and Early Education Star Rated License. North Carolina Department of Health and Human Services. https://ncchildcare.ncdhhs.gov/Services/Licensing/Star-Rated-License.

[31] Centers for Disease Control and Prevention Growth Chart Training. https://www.cdc.gov/nccdphp/dnpao/growthcharts/training/bmiage/index.html.

[32] Carraway-Stage V., Spangler H., Borges M., Goodell L.S. (2014). Evaluation of a pictorial method to assess liking of familiar fruits and vegetables among preschool children. Appetite.

[33] Witt K.E., Dunn C. (2012). Increasing fruit and vegetable consumption among preschoolers: Evaluation of color me healthy. J. Nutr. Educ. Behav..

[34] Sirard J.R., Trost S.G., Pfeiffer K.A., Dowda M., Pate R.R. (2005). Calibration and evaluation of an objective measure of physical activity in preschool children. J. Phys. Act. Health.

[35] Choi L., Ward S.C., Schnelle J.F., Buchowski M.S. (2012). Assessment of wear/nonwear time classification algorithms for triaxial accelerometer. Med. Sci. Sports Exerc..

[36] Shadish W., Cook T.D., Campbell D.T. (2002). Experimental and Quasi-Experimental Designs for Generalized Causal Inference.

[37] Wells N.M., Meyers B.M., Todd L.E., Henderson C.R., Barale K., Gaolach B., Ferenz G., Aitken M., Caroline C.T., Pattison K.O. (2018). The carry-over effects of school gardens on fruit and vegetable availability at home: A randomized controlled trial with low-income elementary schools. Prev. Med..

[38] Evans A., Ranjit N., Rutledge R., Medina J., Jennings R., Smiley A., Stigler M., Hoelscher D. (2012). Exposure to multiple components of a garden-based intervention for middle school students increases fruit and vegetable consumption. Health Promot. Pract..

[39] Tandon P.S., Walters K.M., Igoe B.M., Payne E.C., Johnson D.B. (2017). Physical activity practices, policies and environments in Washington state child care settings: Results of a statewide survey. Matern. Child Health J..

[40] EPA Climate Change Indicators: Length of Growing Seasons. https://www.epa.gov/climate-indicators/climate-change-indicators-length-growing-season.

[41] CCAA (2012). Child Care in America, 2012 State Fact Sheets.

